# Dietary Magnesium Intake and Hyperuricemia among US Adults

**DOI:** 10.3390/nu10030296

**Published:** 2018-03-02

**Authors:** Yiying Zhang, Hongbin Qiu

**Affiliations:** 1 Department of Epidemiology, School of Public Health, Harbin Medical University, Harbin 150081, China; yiyingzhang@hrbmu.edu.cn; 2 School of Public Health, Jiamusi University, Jiamusi 154007, China

**Keywords:** hyperuricemia, magnesium, NHANES, cross-sectional study

## Abstract

To assess the association between dietary magnesium intake and hyperuricemia in United States (US) adults, we extracted 26,796 US adults aged 20–85 years from the National Health and Nutrition Examination Survey (NHANES) in 2001–2014. All dietary intake was measured through 24 h dietary recall method. Multivariable logistic regression analysis was performed to investigate the association between magnesium intake and hyperuricemia after adjusting for several important confounding variables. When compared to the lowest quintile (Q1), for male, adjusted odds ratios (ORs) of hyperuricemia in the second quintile (Q2) to the fifth quintile (Q5) of the magnesium intake were 0.83 (95% CI: 0.72–0.95), 0.74 (0.64–0.85), 0.78 (0.67–0.90), and 0.70 (0.58–0.84, *p* for trend = 0.0003), respectively. For female, OR was 0.75 (0.62–0.90) in the fourth quintile (Q4) (*p* for trend = 0.0242). As compared to Q4 of magnesium intake (contains recommended amount), the relative odds of hyperuricemia were increased by 1.29 times in Q1 (OR = 1.29, 1.11–1.50) in male. The ORs were 1.33 (1.11–1.61) in Q1, 1.27 (1.07–1.50) in Q2 in female. Our results indicated that increased magnesium intake was associated with decreased hyperuricemia risk. It also indicated the importance of recommended dietary allowance (RDA) of magnesium and the potential function of magnesium intake in the prevention of hyperuricemia.

## 1. Introduction

Uric acid is the ultimate product of purine metabolism. When the level of serum uric acid transcends the normal level, hyperuricemia occurs. Previous studies indicated that hyperuricemia not only increased the risk of gout, but also had a close relationship with the development of hypertension, kidney disease, metabolic syndrome, obesity, cardiovascular disease [1,2,3,4,5], lipid metabolism disorders, and type 2 diabetes [6,7]. Nowadays, hyperuricemia is becoming a serious public health problem and epidemiological studies had shown a growing trend in the prevalence of hyperuricemia and gout. The reported prevalence of hyperuricemia ranged from 8.9% to 24.4% in diverse populations [8,9,10,11]. Nevertheless, the pathophysiology of hyperuricemia has not yet been completely illustrated.

Magnesium, which plays a significant role in prevention and treatment of several disorders, is a vital nutrient for human body. Recommended dietary allowance (RDA) of magnesium intake was developed by the Food and Nutrition Board (FNB) of the Institute of Medicine and was based on age and sex. For United States (US) adults, the RDAs for magnesium is 400 mg/day for male aged 19–30, 420 mg/day for male aged 30 and over; 310 mg/day for female aged 19 and 30, and 320 mg/day for female aged 30 and over. RDA is the average daily level of intake sufficient to meet the nutrient requirements of nearly all individuals in a life-stage and gender group [12]. Magnesium insufficiency can lead to many chronic diseases, including cardiovascular disease, type 2 diabetes, osteoporosis, pulmonary disease, depression, migraine headaches, inflammation, and tumors [13]. Many earlier studies proved that dietary magnesium intake is inversely correlated with serum C-reactive protein (CRP) which is an established biomarker of inflammation [14,15,16,17,18]. As hyperuricemia was positively connected with CRP [19,20,21], uric acid may also have a role in inflammation and subsequent inflammatory related diseases [22]. Although magnesium intake may represented an essential and potentially modifiable link to hyperuricemia certain extent, there were only two previous studies investigating the relationship between magnesium intake and hyperuricemia, one revealed a relationship between magnesium deficiency and increased serum uric acid level with 94 diabetic retinopathy patients [23], and another showed that dietary magnesium intake was inversely associated with hyperuricemia in Chinese male [24].

No known studies have explored the association between dietary magnesium intake and hyperuricemia using a nationally representative sample in US. Therefore, the purpose of this cross-sectional study is to assess this correlation in US population with a hypothesis that dietary magnesium intake is inversely correlated with hyperuricemia.

## 2. Materials and Methods

### 2.1. Study Populations

This cross-sectional study used data from the National Health and Nutrition Examination Survey (NHANES), which is a nationally representative survey managed by the Centers for Disease Control and Prevention (CDC) [25]. NHANES is a consecutive survey with every two years, representing one cycle of the US civilian noninstitutionalized population, using a stratified, multistage sampling design. The program covers clinical, physical, laboratory examinations, as well as interviews to get diet and health indicators. NHANES is a publicly available dataset, which resides in the public domain (available on the web at: http://www.cdc.gov/nchs/nhanes.htm). The NCHS Research Ethics Review Board at the National Center for Health Statistics approved the study protocols for NHANES 1999–2016 [26], and additional Institutional Review Board approval for the secondary analyses was not required [27].

A total of 37,215 individuals from 2001 to 2014 aged 20–85 years with uric acid value constituted the study sample. We excluded pregnant women (*n* = 1507) and those with missing essential information on demographic or total nutrient intakes dietary interview (*n* = 8912). After exclusion, 26,796 subjects (13,807 men and 12,989 women) were included in our study.

### 2.2. Study Variables

The major variables included concentrations of uric acid and the intake of magnesium. Serum concentrations of uric acid were detected on a Beckman UniCel^®^ DxC800 Synchron or a Beckman Synchron LX20 (Beckman Coulter, Inc., Brea, CA, USA) after oxidation of uric acid by uricase to form allantoin and H_2_O_2_. Hyperuricemia was defined as serum uric acid ≥7.0 mg/dL in males and ≥6.0 mg/dL in females. The intake of magnesium, energy, and protein were collected from total nutrient intakes provided by the first 24 h dietary recall interview which was obtained in-person in the Mobile Examination Center (MEC). Using estimated amounts of foods, nutrient intakes were computed at the individual-level using a revised nutrient database that converted amounts of specific food intakes into amounts of various nutrients [28], detailed descriptions of the dietary interview methods are provided in the NHANES Dietary Interviewers Procedure Manuals [29]. In addition, factors that had been proved to be correlated with the intake of magnesium as well as hyperuricemia were included in regression models to control for potential confounding. The covariates included age, race/ethnicity, marital status, education background, smoking status, drinking status, body mass index (BMI), waist circumference, hypertension status, diabetes status, energy intake, protein intake, creatinine, gamma glutamyl transferase (GGT), total cholesterol, glucose, triglycerides, and high-density lipoprotein cholesterol (HDL-C). We categorized race/ethnicity as non-Hispanic white, non-Hispanic black, Mexican American, and others (other Hispanics and multi-racial participants). Education background was classified into above high school, high school graduation/General Educational Development (GED), and less than high school. Diabetes status was obtained from self-report. BMI (kg/m^2^) was computed from weight and height. Hypertension was defined as systolic blood pressure ≥140 mm Hg or diastolic blood pressure ≥90 mm Hg.

### 2.3. Statistical Analyses

Respondents were grouped into five levels, according to the magnesium intake quintile: <176 (Q1), 176–234 (Q2), 235–298 (Q3), 299–387 (Q4), and ≥388 mg/day (Q5) for entire respondents; <200 (Q1), 200–265 (Q2), 266–334 (Q3), 335–432 (Q4), and ≥433 mg/day (Q5) for males; <158 (Q1), 158–207 (Q2), 208–260 (Q3), 261–336 (Q4), and ≥337 mg/day (Q5) for females. The continuous variable was presented as median and Inter-Quartile Range (skewed distributed data), and the categorical variable was expressed as percentage. Wilcoxon signed-rank test was used to compare the magnesium intake and the population RDAs (RDAs for magnesium were 400 mg/day for male aged 19–30, 420 mg/day for male aged 30 and over; 310 mg/day for female aged 19 and 30, 320 mg/day for female aged 30 and over [12]). Differences in continuous variable were assessed by the Wilcoxon rank sum test and Kruskal-Wallis H test (non-normally distributed data and heteroscedasticity). Differences in categorical variable were evaluated by the chi-square test and multiple comparisons based on Bonferroni correction. Multivariable logistic regression analysis was used to estimate the odds ratio (OR) and 95% confidence interval (CI) of hyperuricemia, according to the magnesium intake quintile for male and female separately, with the lowest quintile and the fourth quintile being considered as the references, respectively. Models 1 and 4 adjusted for age, race/ethnicity. Based on models 1 and 4, models 2 and 5 further adjusted for smoking status, drinking status, education background, marital status, hypertension status and diabetes status. Models 3 and 6 further adjusted for creatinine, GGT, energy intake, protein intake, total cholesterol, glucose, BMI, waist circumference, HDL-C, and triglycerides. The lowest quintile (Q1) was regarded as the reference in Models 1, 2 and 3. The fourth quintile (Q4 contains the recommended amount) was regarded as the reference in Models 4, 5 and 6. All of the *p* values were two-sided, *p* < 0.05 was regarded as statistically significant, and *p* < 0.0125 (0.05/4), *p* < 0.0167 (0.05/3), *p* < 0.025 (0.05/2) was considered as statistical significance after Bonferroni adjustment for multiple comparisons. All of the analyses were performed using SAS version 9.4 (SAS Institute Inc., Cary, NC, USA).

## 3. Results

The daily magnesium intake was 301 mg (215 mg–414 mg) for male aged 20–30 years, 299 mg (217 mg–400 mg) for male aged 31–85 years, 226 mg (164 mg–306.5 mg) for female aged 20–30 years, and 234 mg (173 mg–314 mg) for female aged 31–85 years, all significantly lower than their respective RDAs [12], as is shown in Table 1. The result of comparing the indicators between hyperuricemia and non-hyperuricemia for both sexes is shown in Table 2. For male, except for marital status (*p* = 0.3895), drinking status (*p* = 0.1323), and diabetes status (*p* = 0.2630), other indicators were all significantly different between hyperuricemia and non-hyperuricemia. Compared to the participants without hyperuricemia, those with hyperuricemia were more likely to be older, non-Hispanic black, former smoker, have hypertension, less likely to be Mexican American, currently smoking, have higher BMI, waist circumference, creatinine, GGT, total cholesterol, glucose, triglycerides, and have lower magnesium intake, energy intake, protein intake, HDL-C. For female, all of the indicators were significantly different between hyperuricemia and non-hyperuricemia. Participants with hyperuricemia were more likely to be older, non-Hispanic black, high school or GED, living alone, never drinking, former drinking, former smoking, have hypertension and diabetes, less likely to be Mexican American, above high school, married or living with partner, currently drinking, never smoking, have higher BMI, waist circumference, creatinine, GGT, total cholesterol, glucose, triglycerides, and have lower energy intake, protein intake, magnesium intake, and HDL-C than normal individuals. More detailed information is presented in Supplementary Table S1.

A description of the characteristics of study participants according to the intake of magnesium is shown in Table 3. Significant differences were detected across all quintiles of magnesium intake for age, gender, race/ethnicity, marital status, education background, smoking status, drinking status, BMI, waist circumference, hypertension status, diabetes status, hyperuricemia, energy intake, protein intake, creatinine, GGT, glucose, triglycerides, and HDL-C. No significant relationship was found between magnesium intake and total cholesterol (*p* = 0.2454). More detailed information is presented in Supplementary Table S2. Participants with higher magnesium intake were more likely to be younger, male, and less likely to be hypertension, diabetes, and hyperuricemia.

The correlation between magnesium intake and hyperuricemia was examined by multivariable model, as is shown in Table 4. The results suggested a strong inverse relationship between magnesium intake and hyperuricemia in this cross-sectional study. Among all participants, the prevalence of hyperuricemia was 20.33%. For male, the prevalence of hyperuricemia was 22.66%, when compared to those consuming less than 200 mg magnesium daily, the relative odds of hyperuricemia were significantly decreased by 0.83 times among those that were consuming 200–265 mg magnesium daily (OR = 0.83, 95% CI: 0.72–0.95), 0.74 times among participants who consumed 266–334 mg daily (OR = 0.74, 95% CI: 0.64–0.85), 0.78 times among those consuming 335–432 mg daily (OR = 0.78, 95% CI: 0.67–0.90), and by 0.70 times among those consuming 433 mg or greater daily (OR = 0.70, 95% CI: 0.58 to 0.84), respectively, and *p* for trend was 0.0003. For female, the prevalence of hyperuricemia was 17.87%, the OR were decreased by 0.75 times among those consuming 261–336 mg magnesium daily (OR = 0.75, 95% CI: 0.62–0.90), when compared to those consuming less than 158 mg daily and *p* for trend was 0.0242. Furthermore, for male, compared to Q4 of magnesium intake (contains recommended amounts), the relative odds of hyperuricemia were increased by 1.29 times in those consuming less than 200 mg magnesium daily (OR = 1.29, 95% CI: 1.11–1.50). For female, the ORs were increased by 1.33 times (OR = 1.33, 95% CI: 1.11–1.61) in those consuming less than 158 mg daily, and by 1.27 times (OR = 1.27, 95% CI: 1.07–1.50) in those consuming 158 to 207 mg daily.

## 4. Discussion

In this cross-sectional study, we found that dietary magnesium intake was inversely associated with hyperuricemia in both male and female among US adults, after adjusting for major confounding factors, including age, race/ethnicity, smoking status, drinking status, education background, marital status, hypertension status, diabetes status, creatinine, GGT, energy intake, protein intake, total cholesterol, glucose, BMI, waist circumference, HDL-C, and triglycerides.

To the best of our knowledge, this is the first and the largest population-based study revealing the relationship between dietary magnesium intake and hyperuricemia in both male and female using a nationally representative sample of US adults. Our findings suggested that magnesium intake was inversely associated with the risk of hyperuricemia. Another similar research in the south India found an inverse correlation between the magnesium and the uric acid level among 94 diabetic retinopathy patients [23]. A cross-sectional study involving 5168 subjects aged 40 years old or above in China has shown a negative association between dietary magnesium intake and hyperuricemia valid for man merely [24], which is different from our result that the inverse association between hyperuricemia and dietary magnesium intake was observed in both men and women. Several factors may account for the difference. Firstly, when compared to previous studies, the sample of our study is the largest (26,796 American adults, including 13,807 men and 12,989 women). Secondly, the participants included women of all ages (20–85 years) and excluded pregnant women, while the subjects in the previous study in China were aged 40 years old or above and the effect of dietary magnesium intake on the exact magnesium level in bodies may be mitigated in middle aged and old women because of their lower serum estrogen level [30]. Thirdly, our study focused on American adults.

Previous studies showed that the dairy product consumption and vitamin C might be helpful in protection against hyperuricemia [31,32,33,34]. The intake of soy products are inversely associated with hyperuricemia [35], vegetable and dairy protein, nuts, legumes, fruits with less sugar, and whole grains would likely lower the risk of gout [36]. Thus, hyperuricemia may be related to dietary modification. Our findings showed that increased magnesium intake may decrease the risks of hyperuricemia. Similarly, magnesium is a component of chlorophyll and green leafy vegetables is an important source. Legumes, fruits, and white vegetables are good dietary sources of magnesium. Besides, nuts, seeds, whole grains, and fortified foods are all rich in magnesium [12,37,38]. In general, foods containing dietary fiber can provide magnesium. Fiber has been identified as being beneficial for intestinal motility and as having a potential act in binding uric acid in the gut for excretion. Therefore, adequate magnesium intake in the diet seems particularly effective in decreasing hyperuricemia risk.

Our results showed that the everyday intake of magnesium in US adults were lower than the recommended amounts, and indicated the importance of RDAs for magnesium: persons with lower intake of magnesium (less than 200 mg per day in male, less than 208 mg per day in female) may have a higher risk of hyperuricemia when compared to those following RDAs of magnesium intake. For males, the RDAs for magnesium ranges from as low as 400 mg/day (age of 19–30 years) to 420 mg/day (age of 30 and over). For females, the RDAs for magnesium ranges from 310 mg/day (age of 19–30 years) to 320 mg/day (age of 30 and over) [12]. Our findings suggested that adequate magnesium intake may have a potential function in the prevention of hyperuricemia, it is beneficial for individuals to keep sufficient magnesium intake, as suggested by RDAs through daily meals to prevent or decrease the risk of hyperuricemia.

The biological mechanism underlying the association between dietary magnesium intake and the prevalence of hyperuricemia was not completely understood, but may be related to the inflammatory mechanism. Laboratory studies have linked magnesium insufficiency to acute inflammatory response mediated by calcium, *N*-methyl-d-aspartate, interleukin-6 (IL-6), and tumor necrosis factor-alpha (TNF-α) [39]. Several epidemiological studies discovered that lower magnesium intake was inversely associated with higher CRP [18,19,20,21,22], which is a well-documented biomarker of inflammation in adults [40,41], children [15], and obese patients [42]. A meta-analysis and systematic review, which included seven cross-sectional studies approved that dietary magnesium intake was inversely correlated with CRP [14]. In addition, many studies reported that hyperuricemia was associated positively with TNF-α, IL-6 and CRP, which suggested that uric acid may have a role in inflammation and subsequent inflammatory related diseases [22,43,44,45]. Moreover, increasing the blood level of uric acid can produce inflammation in the joints and surrounding tissues when crystallized [46]. Further studies are required to investigate the biological mechanism between dietary magnesium intake and hyperuricemia.

In addition, previous studies have shown that growing age and BMI were related to increased hyperuricemia risk, and hypertension may play an independent role for hyperuricemia [10]. Hyperuricemia may also be linked to serum creatinine levels since uric acid had been confirmed to be an influence factor for renal failure [47]. Our study showed that participants with hyperuricemia were more likely to be older, have hypertension, and have higher BMI and creatinine than normal individuals. Previous studies reported that increasing the intake of magnesium in conjunction with taurine can lower blood pressure and improve blood lipid profiles and decrease cardiovascular diseases [48]. Magnesium intake was inversely associated with arterial calcification too, as has been proved in another study [49]. A meta-analysis found that the risk of diabetes decreased when increasing magnesium intake [50]. In our study, participants with higher magnesium intake were less likely to have hypertension and diabetes.

Our study has several strengths. Firstly, this is the first study to assess the relationship between the intake of magnesium and the risk of hyperuricemia among US adults, and use a large (26,796 participants) and nationally representative sample. Secondly, we adjusted for many important confounding variables. Thirdly, the use of trained staff following standardized protocols to measure the basic information of study subjects and conduct interviews improves the precision and efficacy of the data that is obtained.

Our study also has some limitations. First, our study was a cross-sectional study, which limited the definition of the causal correlations, further prospective longitudinal investigations would be important to prove those conclusions. Second, dietary intake levels were estimated from 24 h dietary recall, which may not exactly describe the long-term magnesium intake situation. However, when compared with food frequency questionnaires, 24 h recalls provide greater detail on the varieties and quantities of food eaten and decrease the risk of underestimating or overestimating the intake level of micronutrients. When compared with the blood concentration measurement, blood level may not completely show the nutritional situation, and serum magnesium level only represents less than 1% of the total body magnesium [13]. Finally, further studies are needed to investigate the mechanism of this association.

## 5. Conclusions

Our findings presented a negative correlation between dietary magnesium intake and hyperuricemia in both male and female among US adults after adjusting for major confounding factors. The intake of magnesium of American adults was significantly lower than their respective RDAs. The study indicated the importance of RDAs of magnesium and the potential function of magnesium intake in the prevention of hyperuricemia, and suggested that deficient magnesium intake may increase the risk of hyperuricemia.

## Figures and Tables

**Table 1:** Magnesium intake among United States (US) adults (>19 years) in NHANES 2001–2014.

Age (Years)	RDAs for Magnesium (mg/Day)	Magnesium Intake (mg/Day)	*p*
20–30 ^a^	400.00	301.00 (215.00, 414.00)	<0.0001
31–85 ^a^	420.00	299.00 (217.00, 400.00)	<0.0001
20–30 ^b^	310.00	226.00 (164.00, 306.50)	<0.0001
31–85 ^b^	320.00	234.00 (173.00, 314.00)	<0.0001

^a^ Male; ^b^ Female.

**Table 2:** Characteristics of participants with or without hyperuricemia.

Characteristic	Male		*p*	Female		*p*
	Non-Hyperuricemia (*n* = 10,679)	Hyperuricemia (*n* = 3128)		Non-Hyperuricemia (*n* = 10,668)	Hyperuricemia (*n* = 2321)	
Age (years)	48.00 (34.00, 63.00)	50.00 (35.00, 66.00)	<0.0001	47.00 (34.00, 62.00)	61.00 (48.00, 72.00)	<0.0001
Race/ethnicity (*n*, %)			<0.0001			<0.0001
Non-Hispanic white	5224 (48.92)	1603 (51.25)	0.1846 ^c^	5190 (48.65)	1195 (51.49)	0.1506 ^c^
Non-Hispanic black	1986 (18.60)	698 (22.31)	0.0002 ^c^	1942 (18.20)	602 (25.94)	<0.0001 ^c^
Mexican American	1984 (18.58)	399 (12.76)	<0.0001 ^c^	1872 (17.55)	254 (10.94)	<0.0001 ^c^
Others ^a^	1485 (13.91)	428 (13.68)	0.7824 ^c^	1664 (15.60)	270 (11.63)	<0.0001 ^c^
Education background (*n*, %)			0.0120			<0.0001
>High School	5200 (48.69)	1597 (51.05)	0.1772 ^d^	5709 (53.52)	1081 (46.57)	0.0006 ^d^
High school or GED ^b^	2571 (24.08)	760 (24.30)	0.8423 ^d^	2352 (22.05)	613 (26.41)	0.0004 ^d^
<High School	2908 (27.23)	771 (24.65)	0.0279 ^d^	2607 (24.44)	627 (27.01)	0.0450 ^d^
Marital status (*n*, %)			0.3895			<0.0001
Married or living with partner	7138 (66.84)	2065 (66.02)		5987 (56.12)	1118 (48.17)	0.0001 ^e^
Living alone	3541 (33.16)	1063 (33.98)		4681 (43.88)	1203 (51.83)	<0.0001 ^e^
Drinking status (*n*, %)			0.1323			<0.0001
Never	786 (7.36)	217 (6.94)		2032 (19.05)	522 (22.49)	0.0021 ^d^
Current	8895 (83.29)	2651 (84.75)		6615 (62.01)	1291 (55.62)	0.0043 ^d^
Former	998 (9.35)	260 (8.31)		2021 (18.94)	508 (21.89)	0.0082 ^d^
Smoking status (*n*, %)			<0.0001			<0.0001
Never	4721 (44.21)	1371 (43.83)	0.8153 ^d^	6656 (62.39)	1304 (56.18)	0.0058 ^d^
Current	2823 (26.44)	661 (21.13)	<0.0001 ^d^	2024 (18.97)	384 (16.54)	0.0228 ^d^
Former	3135 (29.36)	1096 (35.04)	<0.0001 ^d^	1988 (18.64)	633 (27.27)	<0.0001 ^d^
Magnesium intake (mg/day)	305.00 (223.00, 408.00)	278.00 (199.00, 380.50)	<0.0001	237.00 (174.00, 317.00)	213.00 (158.00, 289.00)	<0.0001
BMI (kg/m^2^)	27.10 (24.10, 30.44)	29.69 (26.59, 33.66)	<0.0001	27.11 (23.40, 31.91)	31.64 (27.42, 37.10)	<0.0001
Creatinine (mg/dL)	0.97 (0.86, 1.10)	1.04 (0.91, 1.20)	<0.0001	0.73 (0.65, 0.82)	0.90 (0.75, 1.08)	<0.0001
Hypertension status (*n*, %)	2245 (21.02)	877 (28.04)	<0.0001	2109 (19.77)	755 (32.53)	<0.0001
Diabetes status (*n*, %)	1183 (11.08)	369 (11.80)	0.2630	976 (9.15)	492 (21.20)	<0.0001

^a^ Other Hispanics and other races including multi-racial participants; ^b^ General Educational Development; ^c^ Statistically significant after Bonferonni adjustment (0.05/4 = 0.0125); ^d^ Statistically significant after Bonferonni adjustment (0.05/3 = 0.0167); ^e^ Statistically significant after Bonferonni adjustment (0.05/2 = 0.025).

**Table 3:** Characteristics of the participants according to intake of magnesium.

Characteristic	Magnesium Intake (mg/Day)					*p*
	Q1 (<176)	Q2 (176–234)	Q3 (235–298)	Q4 (299–387)	Q5 (≥388)	
	(*n* = 5406)	(*n* = 5394)	(*n* = 5335)	(*n* = 5324)	(*n* = 5337)	
Age (years)	51.00 (34.00, 67.00)	51.00 (36.00, 66.00)	50.00 (35.00, 65.00)	48.50 (35.00, 63.00)	45.00 (33.00, 59.00)	<0.0001
Male (*n*, %)	1935 (35.79)	2281 (42.29)	2654 (49.75)	3102 (58.26)	3835 (71.86)	<0.0001
Race/ethnicity (*n*, %)						<0.0001
Non-Hispanic white	2377 (43.97)	2612 (48.42)	2664 (49.93)	2774 (52.10)	2785 (52.18)	
Non-Hispanic black	1524 (28.19)	1165 (21.60)	957 (17.94)	835 (15.68)	747 (14.00)	
Mexican American	790 (14.61)	815 (15.11)	931 (17.45)	948 (17.81)	1025 (19.21)	
Others ^a^	715 (13.23)	802 (14.87)	783 (14.68)	767 (14.41)	780 (14.61)	
Education background (*n*, %)						<0.0001
>High School	2113 (39.09)	2620 (48.57)	2737 (51.30)	2997 (56.29)	3120 (58.46)	
High school or GED ^b^	1444 (26.71)	1338 (24.81)	1266 (23.73)	1145 (21.51)	1103 (20.67)	
<High School	1849 (34.20)	1436 (26.62)	1332 (24.97)	1182 (22.20)	1114 (20.87)	
Marital status (*n*, %)						<0.0001
Married or living with partner	2921 (54.03)	3177 (58.90)	3313 (62.10)	3432 (64.46)	3465 (64.92)	
Living alone	2485 (45.97)	2217 (41.10)	2022 (37.90)	1892 (35.54)	1872 (35.08)	
Drinking status (*n*, %)						<0.0001
Never	962 (17.80)	826 (15.31)	722 (13.53)	571 (10.73)	476 (8.92)	
Current	3411 (63.10)	3721 (68.98)	3858 (72.31)	4116 (77.31)	4346 (81.43)	
Former	1033 (19.11)	847 (15.70)	755 (14.15)	637 (11.96)	515 (9.65)	
Smoking status (*n*, %)						<0.0001
Never	2776 (51.35)	2909 (53.93)	2891 (54.19)	2797 (52.54)	2679 (50.20)	
Current	1417 (26.21)	1136 (21.06)	1057 (19.81)	1096 (20.59)	1186 (22.22)	
Former	1213 (22.44)	1349 (25.01)	1387 (26.00)	1431 (26.88)	1472 (27.58)	
Hypertension status (*n*, %)	1416 (26.19)	1305 (24.19)	1180 (22.12)	1134 (21.30)	951 (17.82)	<0.0001
Diabetes status (*n*, %)	770 (14.24)	669 (12.40)	566 (10.61)	563 (10.57)	452 (8.47)	<0.0001
Hyperuricemia (*n*, %)	1312 (24.27)	1176 (21.80)	1035 (19.40)	990 (18.60)	936 (17.54)	<0.0001

^a^ Other Hispanics and other races including multi-racial participants; ^b^ General Educational Development.

**Table 4:** Adjusted odds ratios of hyperuricemia among participants associated with magnesium intake.

		Magnesium Intake (mg/Day)					*p* for Trend
		Q1 (<200)	Q2 (200–265)	Q3 (266–334)	Q4 (335–432)	Q5 (≥433)	
		(*n* = 2771)	(*n* = 2753)	(*n* = 2761)	(*n* = 2776)	(*n* = 2746)	
Male (*n* = 13,807)	Model 1 ^a,d^	Reference	0.80 (0.71, 0.90)	0.71 (0.62, 0.80)	0.73 (0.64, 0.82)	0.61 (0.53, 0.69)	<0.0001
	Model 2 ^b,d^	Reference	0.78 (0.69, 0.88)	0.69 (0.61, 0.78)	0.70 (0.62, 0.80)	0.59 (0.51, 0.67)	<0.0001
	Model 3 ^c,d^	Reference	0.83 (0.72, 0.95)	0.74 (0.64, 0.85)	0.78 (0.67, 0.90)	0.70 (0.58, 0.84)	0.0003
	Model 4 ^a,e^	1.38 (1.22, 1.56)	1.10 (0.97, 1.25)	0.97 (0.85, 1.10)	Reference	0.84 (0.73, 0.95)	
	Model 5 ^b,e^	1.43 (1.26, 1.62)	1.11 (0.98, 1.26)	0.98 (0.86, 1.11)	Reference	0.84 (0.73, 0.96)	
	Model 6 ^c,e^	1.29 (1.11, 1.50)	1.07 (0.93, 1.23)	0.95 (0.83, 1.09)	Reference	0.91 (0.78, 1.05)	
		Q1 (<158)	Q2 (158–207)	Q3 (208–260)	Q4 (261–336)	Q5 (≥337)	
		(*n* = 2631)	(*n* = 2625)	(*n* = 2542)	(*n* = 2616)	(*n* = 2575)	
Female (*n* = 12,989)	Model 1 ^a,d^	Reference	0.92 (0.80, 1.06)	0.79 (0.68, 0.91)	0.66 (0.57, 0.76)	0.66 (0.57, 0.77)	<0.0001
	Model 2 ^b,d^	Reference	0.93 (0.81, 1.06)	0.80 (0.70, 0.93)	0.68 (0.58, 0.79)	0.69 (0.59, 0.80)	<0.0001
	Model 3 ^c,d^	Reference	0.95 (0.81, 1.11)	0.86 (0.73, 1.02)	0.75 (0.62, 0.90)	0.87 (0.70, 1.08)	0.0242
	Model 4 ^a,e^	1.52 (1.31, 1.76)	1.40 (1.21, 1.62)	1.20 (1.03, 1.39)	Reference	1.01 (0.86, 1.18)	
	Model 5 ^b,e^	1.48 (1.27, 1.72)	1.37 (1.18, 1.59)	1.19 (1.02, 1.39)	Reference	1.02 (0.87, 1.20)	
	Model 6 ^c,e^	1.33 (1.11, 1.61)	1.27 (1.07, 1.50)	1.15 (0.97, 1.35)	Reference	1.16 (0.98, 1.39)	

^a^ Adjusted for age, race/ethnicity; ^b^ adjusted for age, race/ethnicity, smoking status, drinking status, education background, marital status, hypertension status and diabetes status; ^c^ adjusted for age, race/ethnicity, smoking status, drinking status, education background, marital status, hypertension status, diabetes status, creatinine, gamma glutamyl transferase (GGT), energy intake, protein intake, total cholesterol, glucose, body mass index (BMI), waist circumference, high-density lipoprotein cholesterol (HDL-C) and triglycerides; ^d^ the lowest quintile (Q1) was regarded as the reference; ^e^ the fourth quintile (Q4) was regarded as the reference.

## Supplementary Materials

The following are available online at http://www.mdpi.com/2072-6643/10/3/296/s1, Table S1: Dietary and clinical characteristics of participants with and without hyperuricemia, Table S2: Dietary and clinical characteristics of the participants according to intake of magnesium.
