# Supplementary material for: Dietary Magnesium Intake and Hyperuricemia among US Adults
Source: Nutrients. 2018 Mar 2;10(3):296. doi: 10.3390/nu10030296 (PMC5872714; doi:10.3390/nu10030296)
Supplement: Supplementary file 1 [file nutrients-10-00296-s001.pdf]

Table S1. Dietary and clinical characteristics of participants with and without hyperuricemia.

Characteristic	Male			Female		
	**Non-hyperuricemia (*n* = 10679)**	**Hyperuricemia (*n* = 3128)**	***p***	**Non-hyperuricemia (*n* = 10668)**	**Hyperuricemia (*n* = 2321)**	***p***
Energy intake (kcal/day)	2331.00 (1729.00, 3045.00)	2175.50 (1616.50, 2878.00)	<.0001	1702.50 (1293.00, 2195.50)	1545.00 (1176.00, 2028.00)	<.0001
Protein intake (gm/day)	88.02 (63.88, 118.63)	83.19 (59.15, 114.35)	<.0001	63.29 (46.31, 84.48)	59.85 (42.82, 78.97)	<.0001
Waist circumference (cm)	97.80 (88.90, 107.40)	105.90 (97.00, 115.65)	<.0001	92.55 (83.00, 103.30)	105.00 (94.90, 115.10)	<.0001
GGT (U/L)	23.00 (16.00, 34.00)	29.00 (20.00, 44.00)	<.0001	16.00 (12.00, 24.00)	21.00 (16.00, 33.00)	<.0001
Total cholesterol (mg/dL)	189.00 (163.00, 217.00)	196.00 (168.00, 225.00)	<.0001	195.00 (170.00, 223.00)	201.00 (175.00, 233.00)	<.0001
Glucose (mg/dL)	93.00 (86.00, 104.00)	96.00 (88.00, 108.00)	<.0001	90.00 (83.00, 99.00)	97.00 (88.00, 112.00)	<.0001
Triglycerides (mg/dL)	121.00 (80.00, 191.00)	152.00 (101.50, 235.00)	<.0001	105.00 (72.00, 157.00)	145.00 (100.00, 212.00)	<.0001
HDL-C (mg/dL)	46.00 (39.00, 56.00)	43.00 (36.00, 51.00)	<.0001	56.00 (47.00, 68.00)	51.00 (42.00, 62.00)	<.0001

Table S2. Dietary and clinical characteristics of the participants according to intake of magnesium.

Characteristic	Magnesium Intake(mg/day)					*p*
	Q1(<176) (*n* = 5406)	Q2(176-234) (*n* = 5394)	Q3(235-298) (*n* = 5335)	Q4(299-387) (*n* = 5324)	Q5(≥388) (*n* = 5337)	
Energy intake (kcal/day)	1212.00(916.00, 1550.00)	1648.00(1333.00, 2024.00)	1968.00(1587.00, 2416.00)	2301.00(1883.50, 2846.00)	3008.00(2389.00, 3789.00)	<.0001
Protein intake (gm/day)	43.54(31.07, 57.12)	61.32(48.68, 78.01)	75.76(59.35, 93.46)	89.22(71.25, 111.73)	116.69(91.03, 150.39)	<.0001
BMI (kg/m^2)	28.24(24.40, 32.90)	27.92(24.36, 32.21)	27.95(24.34, 32.10)	27.54(24.22, 31.54)	27.20(24.00, 31.11)	<.0001
Waist circumference (cm)	98.10(87.40, 109.00)	97.70(87.50, 108.00)	97.50(88.00, 108.30)	97.20(87.40, 107.60)	96.50(87.20, 107.00)	0.0029
Creatinine (mg/dL)	0.84(0.70, 1.01)	0.86(0.72, 1.00)	0.87(0.72, 1.00)	0.90(0.73, 1.01)	0.90(0.79, 1.02)	<.0001
GGT (U/L)	20.00(14.00, 30.00)	20.00(14.00, 31.00)	20.00(14.00, 31.00)	21.00(15.00, 32.00)	21.00(15.00, 32.00)	<.0001
Total cholesterol (mg/dL)	193.00(166.00, 222.00)	194.00(168.00, 222.00)	194.00(168.00, 221.00)	193.00(167.00, 221.00)	193.00(167.00, 221.00)	0.2454
Glucose (mg/dL)	92.00(85.00, 104.00)	93.00(85.00, 104.00)	92.00(85.00, 103.00)	92.00(85.00, 103.00)	92.00(85.00, 101.00)	<.0001
Triglycerides (mg/dL)	117.00(78.00, 175.00)	117.00(79.00, 180.00)	121.00(80.00, 183.00)	122.00(80.00, 190.00)	120.00(79.00, 192.00)	0.0001
HDL-C (mg/dL)	50.00(41.00, 61.00)	51.00(42.00, 62.00)	51.00(42.00, 62.00)	50.00(41.00, 61.00)	49.00(41.00, 61.00)	<.0001
